# Instrumental variables in real‐world clinical studies of dementia and neurodegenerative disease: Systematic review of the subject‐matter argumentation, falsification test, and study design strategies to justify a valid instrument

**DOI:** 10.1002/brb3.3371

**Published:** 2024-01-06

**Authors:** Shaun Hiu, Tingting Yong, Jahfer Hasoon, M. Dawn Teare, John‐Paul Taylor, Nan Lin

**Affiliations:** ^1^ Biostatistics Research Group, Population Health Sciences Institute Newcastle University Newcastle upon Tyne UK; ^2^ Cumbria, Northumberland Tyne and Wear NHS Foundation Trust Newcastle upon Tyne UK; ^3^ Translational and Clinical Research Institute, Campus for Ageing and Vitality Newcastle University Newcastle upon Tyne UK

**Keywords:** dementia, epidemiology, instrumental variables, neurodegenerative disease, systematic review

## Abstract

**Objectives:**

We systematically reviewed how investigators argued for and justified the validity of their instrumental variables (IV) in clinical studies of dementia and neurodegenerative disease.

**Methods:**

We included studies using IV analysis with observational data to investigate causal effects in clinical research studies of dementia and neurodegenerative disease. We reported the subject‐matter argumentation, falsification test, and study design strategies used to satisfy the three assumptions of a valid IV: relevance, exclusion restriction, and exchangeability.

**Results:**

Justification for the relevance assumption was performed in all 12 included studies, exclusion restriction in seven studies, and exchangeability in nine studies. Two subject‐matter argumentation strategies emerged from seven studies on the relevance of their IV. All studies except one provided quantitative evidence for the strength of the association between the IV and exposure variable. Four argumentation strategies emerged for exclusion restriction from six studies. Four falsification tests were performed across three studies. Three argumentation strategies emerged for exchangeability across four studies. Nine falsification tests were performed across nine studies. Two notable study design strategies were reported.

**Conclusion:**

Our results reinforce IV analysis as a feasible option for clinical researchers in dementia and neurodegenerative disease by clarifying known strategies used to validate an IV.

## Graphical abstract

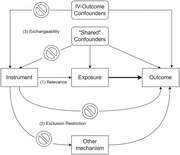

## 1 INTRODUCTION

Longitudinal research cohorts and routinely collected electronic health data are now increasingly being used to study the causal effects of therapies and public health programs in lieu of clinical trials. The fields of dementia and neurodegenerative disease have benefitted from causal inference methods applied to drug‐repurposing research, risk profile of medications, and the design of potential confirmatory trials (Ahn et al., 2022; Caniglia et al., 2020; Charpignon et al., 2022). However, causal inference methods are often rest on several unverifiable, though partially empirically testable, assumptions; the violation of which may harm the credibility of causal claims.

In this systematic review, we focus on a particular causal inference method—instrumental variable (IV) analysis. The primary appeal of IV analysis is that it allows one to identify causal effects of the exposure on the outcome even when there is unmeasured confounding of the exposure‐outcome relationship (Baiocchi et al., 2014). This overcomes a limitation of other causal inference methods such as propensity score based methods which only allow for the control of measured confounders.

**FIGURE 1:**
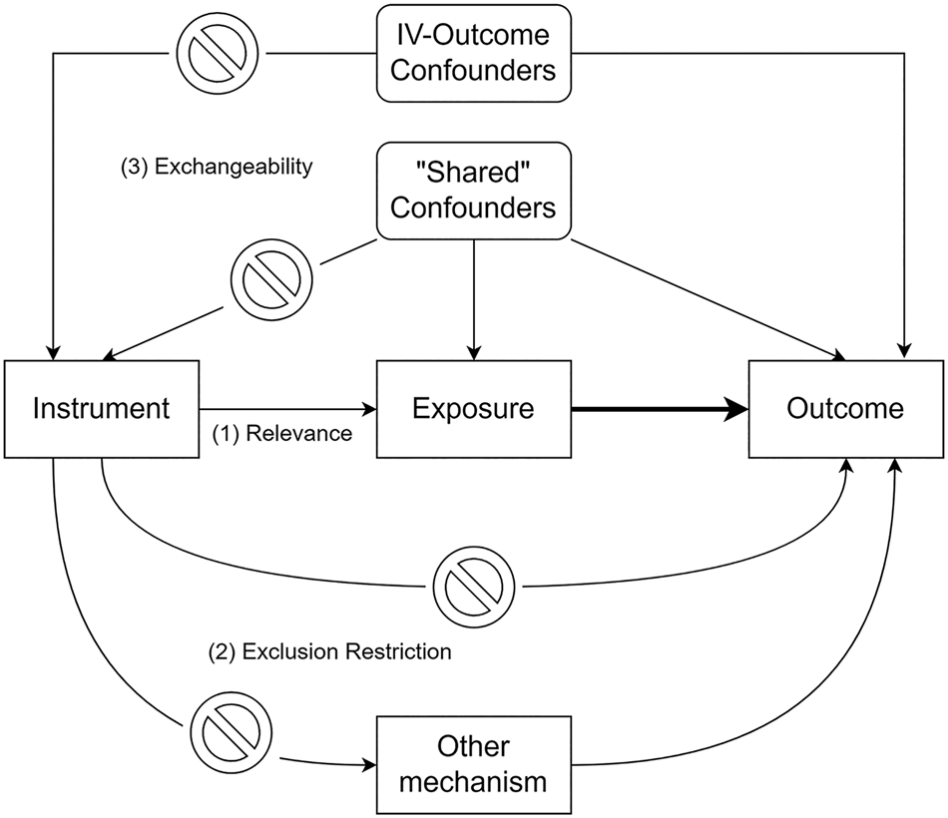
Visual summary of the three instrumental variable assumptions. Figure illustrates the three instrumental variable assumptions in a causal diagram. A valid instrumental variable (IV) is one that satisfies three assumptions: (1) relevance—the IV influences the exposure of interest; (2) exclusion restriction—the IV has an effect on the outcome only via the exposure and no other mechanism/pathway (whether directly or indirectly); and (3) exchangeability—the IV does not share common causes with the outcome. Arrows indicate the path and direction of the causal effect—with an implication that the causal direction goes forward in time. The bolded arrow between exposure and outcome indicates the causal effect of interest. Stop symbols indicate that the assumptions require that certain paths be blocked through modeling or by design in order to identify causal effects.

Central to the methodology is the identification of a valid instrument—one which functions as a source of natural random variation that mimics the effects of randomization seen in clinical trials (Widding‐Havneraas & Zachrisson, 2022). A valid IV is one that satisfies three assumptions: (1) relevance; (2) exclusion restriction; and (3) exchangeability (see Figure 1 for more detail) (Labrecque & Swanson, 2018; Lousdal, 2018). Support for each of the three assumptions requires different types of evidence and reasoning. Relevance is empirically justifiable usually by quantifying the association (or strength) between the IV and exposure (Davies et al., 2013), though it may sometimes be supplemented and/or complemented with subject‐matter knowledge (Chen & Briesacher, 2011). Exclusion restriction and exchangeability on the other hand require subject‐matter knowledge and convincing argumentation. Arguments can be made based on those citing prior knowledge and careful reasoning (subject‐matter argumentation) and/or failures to falsify the validity of an IV (falsification tests). Falsification tests are quantitative tests and assessments that aim to cast doubt on the assumptions. Though these tests do not directly prove that an assumption holds, failing a falsification test may provide evidence that the assumptions are at least implausible (Keele et al., 2019). Certain design decisions may also improve the credibility of an instrument's validity for example by restricting the population to a known subgroup for whom the IV assumptions are likely to apply (Baiocchi et al., 2014).

**FIGURE 2:**
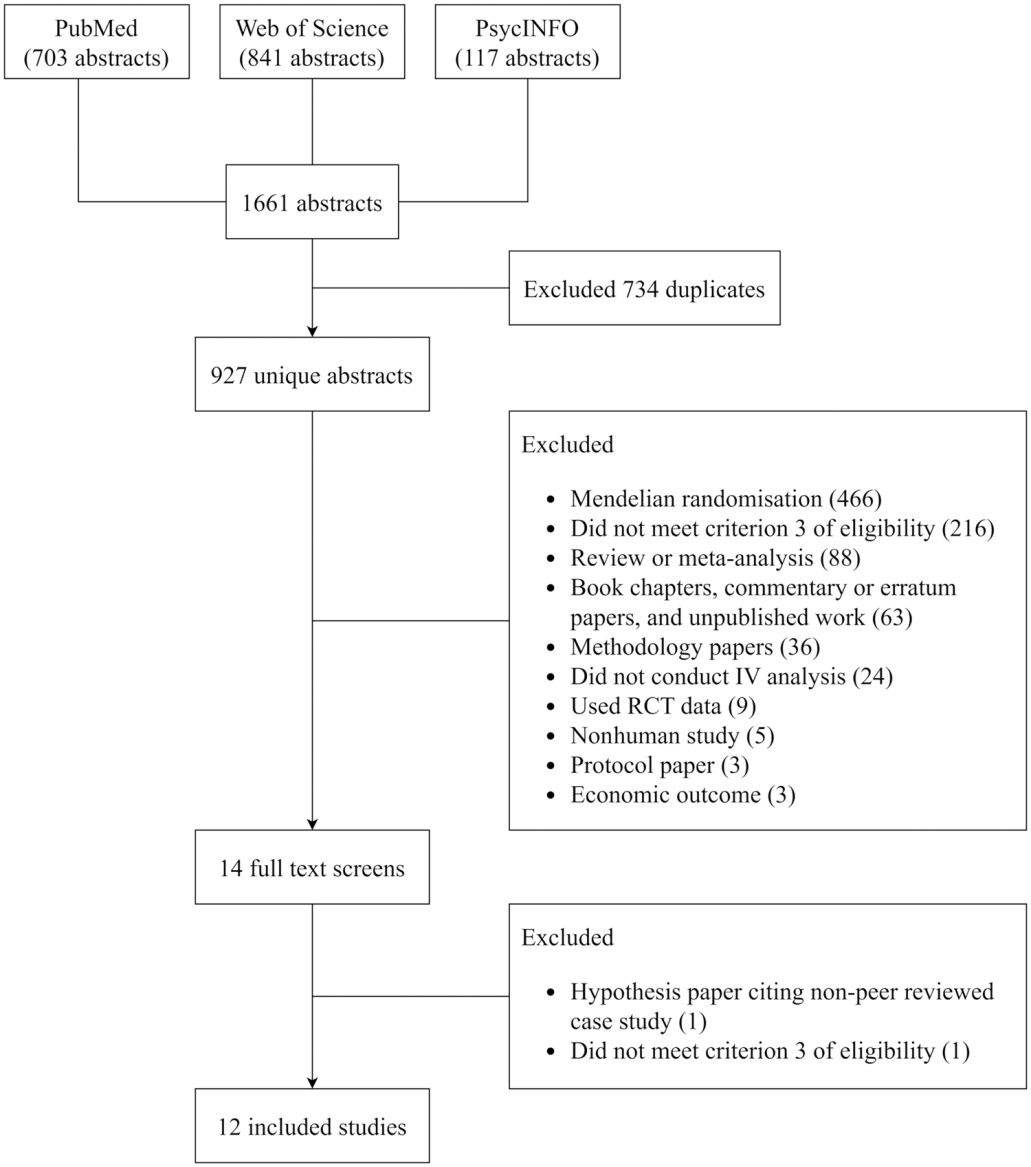
Flow chart of study inclusion.

The objective of this review was to build a landscape of how clinical studies of dementia and neurodegenerative disease have justified the validity of their IVs. We reviewed the subject‐matter argumentation, falsification test, and study design strategies used and inform practical recommendations. We additionally impart a set of tools to assist in the critical appraisal of an IV. Our focus on clinical studies excludes Mendelian randomization (MR) studies, which are IV studies that use genetic instruments. This decision was in response to our observations that IV analysis may be underutilized in clinical research of dementia and neurodegenerative disease research; we observed a much greater uptake of IV methods in preclinical dementia and neurodegenerative disease research—even though IV analysis is not exclusive to either (Figure 2). The main drivers of the underutilization are perhaps the lack of understanding of the IV approach in clinical research of dementia and neurodegenerative disease, particularly over how to justify a valid IV. This may reflect that MR is relatively well established in preclinical research with accepted frameworks and toolkits, and IV assumptions like exchangeability may be more straightforwardly justified with genetic instruments because of Mendel's laws of inheritance (Sanderson et al., 2022). Our review seeks to help create awareness and reinforce IV analysis as a feasible option for clinical researchers in dementia and neurodegenerative disease to investigate causal effects.

## 2 MATERIALS AND METHODS

### 2.1 Search strategy

The review protocol was registered on PROSPERO (CRD42023392589). We amended our study to exclude MR studies to focus on IV analysis in clinical settings. We also relaxed the inclusion criteria to include outcome measures of dementia symptom staging or dementia risk score; these outcomes were deemed clinically relevant and their inclusion would allow us to cover studies that may not have access to or collected diagnosis data. The review adhered to the Preferred Reporting Items for Systematic Reviews and Meta‐Analysis guidelines (Page et al., 2021).

We conducted our search using PubMed, PsycINFO, and Web of Science and covered publications prior to January 20 2023. The search terms are reported in Supporting Information 1. Studies were screened for eligibility on Rayyan by two study authors (SH and TY) (Ouzzani et al., 2016).

Inclusion criteria were as follows:

1. Published articles in English using IV analysis; and
2. Used observational data from an adult population (18+); and
3. Investigated the causal effect of
  - Any exposure on risk of dementia, major neurocognitive disorder, or neurodegenerative disease; or
  - Dementia, major neurocognitive disorder, or neurodegenerative disease on a set of clinical outcomes of interest (Supporting Information 1); or
  - Any exposure on a set of clinical outcomes of interest within patients diagnosed with dementia, major neurocognitive disorder, or neurodegenerative disease.

Reviews, meta‐analyses, commentary, or papers emphasizing statistical/methodological research, conference abstracts, unpublished work, protocols, studies using clustered units of observation (e.g., country‐level units), studies reporting economic outcomes only, studies using clinical trial data only, and MR studies were excluded.

### 2.2 Data collection, synthesis, and reporting

Data related to assumptions were extracted by two authors (SH and TY) and reviewed by another author (JH). The following were extracted and reported descriptively:

- Study characteristics;
- Instrumental variable(s), exposure(s), and outcome(s);
- Test statistic reported showing support of the relevance assumption;
- Text data relevant to subject‐matter argumentation, falsification tests, and study designs supporting the IV assumptions.

One author (SH) coded the text data into subject‐matter argumentation and falsification test descriptors and developed an initial code framework (Braun & Clarke, 2013). The coded data and descriptors were reviewed by all authors. Disagreements were resolved by consensus.

We quantified the degree to which a study argued for the validity of its IV. We adapted and expanded on a two‐point rating scale by Chen and Briesacher (2011) and developed a five‐point “IV validation appraisal” scale. One point was awarded if a study: (i) provided empirical evidence or a theoretical reason for the relevance assumption; (ii) made a subject‐matter argument for the exclusion restriction assumption; (iii) conducted at least one falsification test to support the exclusion restriction assumption; (iv) made a subject‐matter argument for the exchangeability assumption; or (v) conducted at least one falsification test to support the exchangeability assumption. Our scale should not be interpreted as a validated scale but as a tool to help the skeptical reader critically appraise the IV justification process.

## 3 RESULTS

We identified 927 unique publications from our search and 12 studies met our eligibility criteria (Burke et al., 2022; Hebert et al., 2013; Hikichi et al., 2016; Jayadevappa et al., 2019; Joyce et al., 2018; Lei et al., 2020; Lind et al., 2021; Nguyen et al., 2016; Reynolds et al., 2020; Sato et al., 2021; Thunell et al., 2022; Walker et al., 2020) (Figure 2). We excluded 466 MR studies during screening and this informed the focus of our research. The study characteristics are summarized collectively in Table 1 and by each study in Table 2. Data mostly originated from routinely collected sources such as administrative claims data in three geographic locations—Japan, the United Kingdom, and the United States. All studies, with the exception of two, used a single IV in their modeling. The majority of instruments were based on a feature of geographical location (e.g., treatment rates). Three studies investigated a pharmacological exposure. The types of outcomes reported across all studies were varied but most either investigated incident dementia or hospital (re)admission. Four studies reported multiple outcomes.

**TABLE 1:** Summary characteristics of all included studies.

	Included studies (*n* = 12)
**Publication year, *n* (%)**	
2013	1 (8)
2016	2 (17)
2018	1 (8)
2019	1 (8)
2020	3 (25)
2021	2 (17)
2022	2 (17)
**Sample size, median (range)**	135,554 (3,566–3,333,617)
**Primary source of data, *n* (%)**	
Routinely collected electronic health data	9 (75)
Longitudinal research cohort data	3 (25)
**Country, *n* (%)**	
Japan	2 (17)
United Kingdom	1 (8)
United States	9 (75)
**Target population, *n* (%)** ^a^	
Older adults (≥65) without dementia	4 (33)
Older adults without dementia and diagnosed with prostate cancer	1 (8)
Older adults with dementia	4 (33)
Older adults with PD	1 (8)
Older adults (dementia status unspecified)	1 (8)
Adults over 50 (dementia status unspecified)	1 (8)
Adults over 40 without dementia	1 (8)
**Type of instrumental variable, *n* (%)** ^b^	
*Single instrument*	
Geographic distance	4 (33)
Geographic treatment rates	3 (25)
Physician preference	3 (25)
Geographic climate	1 (8)
*Multiple instruments*	
Geographic schooling policies and characteristics	1 (8)
Geographic treatment rates	1 (8)
**Types of exposure, *n* (%)**	
Pharmacological	3 (25)
Non‐pharmacological	9 (75)
**Types of outcomes reported, *n* (%)**	
Incident dementia	6 (50)
Hospital admission or readmission	3 (25)
Mortality	1 (8)
Dementia probability score	1 (8)
Change in dementia symptom severity	1 (8)
Inappropriate antipsychotic use	1 (8)
Use of physical restraints	1 (8)
Use of feeding tubes	1 (8)
Use of indwelling urinary catheters	1 (8)
Presence of pressure ulcers	1 (8)
Medication adherence	1 (8)
Subsequent neuropsychological testing	1 (8)

*Note*: Studies may report more than one outcome.

Abbreviations: AD, Alzheimer's disease; PD, Parkinson's disease.

^a^ Reynolds et al. (2020) analyzed their dementia and PD patient subgroups separately.

^b^ Hebert et al. (2013) used two instruments separately in different models (one based on physician preference and one based on geographic treatment rates).

**TABLE 2:** Line listing of characteristics of each included study.

References	Country	Data sources	Sample size	Type of IV(s)	Operationalization of IV(s)	Exposure/treatment of interest	Comparator (if binary exposure)	Outcome(s)
Burke et al. (2022)	United States	Medicare (MedPAR, MDS, OASIS, MBSF)	977,946	Geographic distance	Dichotomization of differential distance from patient residence to nearest home health or skilled nursing facility	Posthospital discharge to a home health facility	Posthospital discharge to a skilled nursing facility	1. Unplanned hospital readmission within 30 days 2. 30‐day mortality 3. 100‐day mortality 4. Composite of 1 and 2
Hebert et al. (2013)	United States	Medicare (enhanced 5% random sample of beneficiaries)	New users of ACEIs: 9,840 Prevalent users of ACEIs: 107,179	1. Physician preference 2. Geographic treatment rates	Tested two IVs separately: 1. Type of ACEI prescribed to prior patient 2. Ratio between observed and expected centrally‐active ACEI geographic prescription rates	Centrally active ACEIs	Non‐centrally active ACEIs	Time to incident ADRD
Hikichi et al. (2016)	Japan	JAGES data with linkage to national LTCI registry	3,566	Geographic distance	Inverse distance from coastline to participant residence	Self‐reported extent of housing damage	–	Standardized in‐home assessment of dementia symptomology
Jayadevappa et al. (2019)	United States	SEER‐Medicare linked database	154,089	Geographic treatment rates	Dichotomization of geographic rates of patients treated with androgen deprivation therapy	Androgen deprivation therapy	No androgen deprivation therapy	Time to incident AD or dementia
Joyce et al. (2018)	United States	Medicare (MedPAR, MDS, OSCAR)	704,782	Geographic distance	Differential distance from the patient residence to nursing home with dementia special care unit relative to one without	Admission to nursing home with dementia special care unit	Admission to nursing home without dementia special care unit	1. Inappropriate antipsychotic use 2. Use of physical restraints 3. Use of feeding tubes 4. Use of indwelling urinary catheters 5. Presence of pressure ulcers 6. Hospitalization
Lei et al. (2020)	United States	VHA Office of Geriatrics & Extended Care Data Analysis Center Core Files and Medicare (MDS, Medicare Carrier Standard Analytic Files)	105,528	Geographic distance	Change of residence by more than 10 miles	Bice‐Boxerman Continuity of Care index	–	1. All‐cause hospitalization 2. Hospitalization for ambulatory care sensitive condition 3. Hospitalization for a number of major diagnostic categories
Lind et al. (2021)	United States	Medicare (LDS 5% random sample) and HRSA Area Health Resources File	324,485	Geographic treatment rates	Multiple instruments: County‐level, ethnicity‐specific (White, Black, Asian, and Hispanic) “Welcome to Medicare” (WMV) utilization rates	Received Medicare Annual Wellness Visit	Did not receive Medicare Annual Wellness Visit	Incident dementia Subsequent neuropsychological testing
Nguyen et al. (2016)	United States	The Health and Retirement Study	10,955	Geographic schooling policies and characteristics	Multiple instruments^a^	Self‐reported years of schooling	–	Dementia probability score
Reynolds et al. (2020)	United States	Clinformatics DataMart database	Patients with neuropathy on gabapentinoids: 52,249 Patients with neuropathy on SNRIs: 5,246 Patients with dementia on cholinesterase inhibitors: 19,820 Patients with PD on dopamine agonists: 3,130	Physician preference	Physician's “choice of individual medications for the same condition with similar efficacy and tolerability” (page e1417)	Out of pocket costs associated with gabapentinoids (for neuropathy subgroup), cholinesterase inhibitors (for dementia subgroup), and dopamine agonists (for PD subgroup)	–	Medication adherence in first 6 months of first prescription
Sato et al. (2021)	Japan	JAGES data with linkage to national LTCI registry	73,260	Geographic climate	Residence in a snowy region	Physical activity index	–	Incident dementia
Thunell et al. (2022)	United States	Medicare (20% random sample of beneficiaries)	3,333,617	Geographic treatment rates	Change in rate of county‐level Medicare Annual Wellness Visit utilization compared to the prior year	Received Medicare Annual Wellness Visit	Did not receive Medicare Annual Wellness Visit	Incident ADRD or mild cognitive impairment
Walker et al. (2020)	United Kingdom	CPRD‐GOLD	849,378	Physician preference	Number of prescriptions of each antihypertensive drug class from seven most recent patients prescribed with antihypertensive medication	Pairwise comparisons of 1–7 against other antihypertensive classes 1. α‐adrenoceptor blockers 2. Angiotensin‐converting enzyme inhibitors 3. Angiotensin II receptor blockers 4. β‐adrenoceptor blockers 5. Calcium‐channel blockers 6. Diuretics 7. Vasodilator antihypertensives	Other antihypertensive drug classes	Incident dementia

Abbreviations: ACEI, Angiotensin‐converting enzyme inhibitors; AD, Alzheimer's disease; ADRD, Alzheimer's disease and related dementias; CPRD, Clinical Practice Research Datalink; HRSA, Health Resources & Services Administration; IV, Instrumental variable; JAGES, Japan Gerontological Evaluation Study; LDS, limited dataset; LTCI, long‐term care insurance; MBSF, Medicare Master Beneficiary Summary Files; MDS, minimum dataset; MedPAR, Medicare Provider Analysis and Review; OASIS, Outcome and Assessment Information Set; OSCAR, Online Survey, Certification, and Reporting; PD, Parkinson's disease; SEER, Surveillance, Epidemiology, and End Results; SNRI, serotonin and norepinephrine reuptake inhibitors; VHA, Veterans Health Administration.

^a^ Due to the detailed definitions, we refer readers to the “Compulsory schooling laws and school characteristics” subsection of Nguyen et al. (2016, page 72).

### 3.1 Appraisal of IV validation process

The strategies each study used to help meet the IV assumptions are summarized in Table 3. The extracted verbatim text data used to create the subject‐matter argument codes are presented in Supporting Information 2. All studies scored at least one point on the IV validation appraisal score (Table 3). Whether by subject‐matter arguments or falsification testing, justification for the relevance assumption was performed by all studies, exclusion restriction in seven studies, and exchangeability in nine studies. Most studies scored three points on the IV validation appraisal score (*n* = 6). One study obtained the maximum score.

**TABLE 3:** Strategies to support validity of instrument with instrumental variable (IV) validity appraisal score.

	Relevance (0–1 point)		Exclusion restriction (0–2 points)		Exchangeability (0–2 points)		IV validity appraisal
References	Empirical evidence	Reason	Subject‐matter argument	Falsification test	Subject‐matter argument	Falsification test	Score
Burke et al. (2022)	*F*‐statistic	Prior quantitative or qualitative evidence Explanation of mechanism how IV influences exposure			Provision of mechanism why unmeasured confounding is unlikely	Inspection of baseline characteristics across IV levels	3
Hebert et al. (2013)	Proportion of treated within IV levels				Unmeasured confounding unlikely due to nature of study period Unmeasured confounding unlikely due to the required complexity Provision of mechanism why unmeasured confounding is unlikely	Inspection of baseline characteristics across IV levels Baseline hypothesis testing	3
Hikichi et al. (2016)	*F*‐statistic			Adjust for exposure and evaluate statistical significance of IV^a^			2
Jayadevappa et al. (2019)	*F* statistic (but unreported)						1
Joyce et al. (2018)	*F*‐statistic	Prior quantitative or qualitative evidence			Provision of mechanism why unmeasured confounding is unlikely	Inspection of baseline characteristics across IV levels Baseline hypothesis testing Standardized mean differences	3
Lei et al. (2020)	Partial *F*	Explanation of mechanism how IV influences exposure				Standardized mean differences Inspection of baseline characteristics across IV levels Population stratification	2
Lind et al. (2021)	*F*‐statistic	Explanation of mechanism how IV influences exposure	Other mechanisms unlikely due to nature of outcome			Negative control outcome	3
Nguyen et al. (2016)	*F*‐statistic Partial *R* ^2^		Other mechanisms unlikely due to nature of IV	Adjust for exposure and evaluate sign of IV effect estimate Analysis in subgroup where IV does not cause exposure		Analysis in subgroup where IV does not cause exposure	4
Reynolds et al. (2020)		Prior quantitative or qualitative evidence	Reasonable assumption given nature of exposure				2
Sato et al. (2021)	Partial *F*	Explanation of mechanism how IV influences exposure	Other mechanisms unlikely due to nature of IV			Inspection of baseline characteristics across IV levels	3
Thunell et al. (2022)	*F*‐statistic Correlation between IV and Exposure		Other mechanisms unlikely due to nature of IV			Negative control outcome Population stratification	3
Walker et al. (2020)	Partial *F*	Prior quantitative or qualitative evidence Bonet's instrumental inequality test	Other mechanisms unlikely but without further elaboration	Bonet's instrumental inequality test	Unmeasured confounding implausible due to nature of study period Provision of mechanism why unmeasured confounding implausible	Bias components plots Bonet's instrumental inequality test Sargan–Hansen overidentification test	5

^a^ Argued to be an invalid falsification test by Baiocchi et al. (2014).

### 3.2 Relevance

Two argumentation strategies emerged from seven studies that provided subject‐matter arguments on the relevance of their IV. The first strategy involved citing prior quantitative and/or qualitative studies (*n* = 4), and the second involved the provision of an explanation on the mechanism by which the instrument influenced the exposure/treatment (*n* = 4).

Regarding empirical evidence, all studies except one provided quantitative evidence for the strength of the association between the IV and exposure variable. Most evidence of associations were in the form of the *F*‐statistic (*n* = 7), followed by partial *F* (*n* = 3), partial *R* ^2^ (*n* = 1), Pearson's correlation coefficient (*n* = 1), or a hypothesis test comparing the proportion of those exposed across levels of the IV (*n* = 1). Of the 11 studies that provided quantitative evidence, one did not report the value of their statistic.

Three falsification tests were performed across five studies. Two studies that categorized a continuous IV for analysis tested various cut‐offs to check that results were not driven by a particular subgroup (*n* = 2). Two studies using IVs based on geographic distance tested for associations between the IV and exposure in subgroups of participants for whom the instrument was not expected to influence the exposure (*n* = 2). For example, Burke et al. (2022) examined the strength of their instrument (differential distance from residential address to nearest type of post‐acute care facility) in a subgroup of patients whose residential address was very distal from the admitting hospital. This was done to provide evidence that because the patient lived far from the hospital, the type of post‐acute care referred would be decided almost independently from whether the patient lived closer to one facility type than another. Bonet's instrumental inequality test was used in one study to test if their IV was a valid instrument (Walker et al., 2020). The inequality test states that if the IV is valid, then the conditional distribution of the exposure and outcome, given the IV, should adhere to a certain constraint (Bonet, 2001). The inequality test provides a joint test of all three assumptions; failing the inequality test suggests that one or more of the assumptions are violated. This instrumental inequality is a necessary property for an IV to be valid, but it is not sufficient unless the exposure is binary (Pearl, 1995). Furthermore, the inequality test is applicable only when the exposure is discrete.

### 3.3 Exclusion restriction

Four argumentation strategies emerged for exclusion restriction across six studies. The first strategy involved arguing that, due to nature of the outcome, it was unlikely that the IV affected the outcome by any other mechanism other than the exposure (*n* = 1). For example, Lind et al. (2021) were concerned that areas with high rates of Medicare Welcome to Medicare Visits and Annual Wellness Visits may improve in their delivery of preventive care over the course of their study timeframe, which may influence patient health. However, they argued that this was unlikely due to the length it takes on average to develop dementia. The second was a variation of the first strategy but due to nature of the IV instead (*n* = 3). As an example, Sato et al. (2021) argued that it was challenging to hypothesize a plausible mechanism by which residence in a high snowfall area would directly lead to dementia risk. The third strategy involved arguing that the assumption was reasonable given nature of the exposure (*n* = 1). Reynolds et al. (2020) posited that, by the nature of their chosen medications under investigation, efficacy and tolerability would be the primary mechanism by which physician preference would influence adherence. The last strategy involved arguing that it was unlikely that there would be other pathways from the IV to the outcome except via the exposure but without further elaboration (*n* = 1; see Supporting Information 2) (Walker et al., 2020).

Four falsification tests were performed across three studies. Two studies regressed the outcome on the IV while adjusting for the exposure and covariates in the same model to test the assumption, but with different criteria for falsification. Hikichi et al. (2016) evaluated the statistical significance of the IV on the outcome, whereas Nguyen et al. (2016) evaluated the sign of their estimated effect of the IV by incorporating prior knowledge in the form of a causal diagram. The latter study first assumed the presence of unmeasured confounding between the exposure and outcome, what the unmeasured confounders might have been, and leveraged prior knowledge on the signs of the effects the unmeasured confounders would likely have had on the exposure and outcome. Under the assumption that there was such an unmeasured variable that was positively associated with the exposure but negatively associated with the outcome, they hypothesized that if the exposure was adjusted for in the model, then either the sign of the effect of the IV on the outcome would be positive or there would be a null effect. Nguyen et al. (2016) also attempted to falsify the assumption by repeating their analysis in a subgroup for which the instrument does not influence the exposure. The rationale was that the only possible way the IV would have an association with the outcome in this subgroup was either through an alternative mechanism or an unmeasured confounder(s); in other words, a falsification test for exclusion restriction and exchangeability (Baiocchi et al., 2014). Walker et al. (2020) used Bonet's instrumental inequality test to test if their IV was a valid instrument, which has been described in the relevance section.

### 3.4 Exchangeability

Three argumentation strategies emerged for exchangeability across four studies. The first strategy involved making the argument that unmeasured confounding was implausible due to the study period (*n* = 2). For example, Walker et al. (2020) argued that by restricting their study period, it was unlikely that patient characteristics could confound their IV (physician preference) and incident dementia as prior to year 2015 it was a requirement for patients in the United Kingdom to live within a general practitioner's boundary area in order to register. Thus, the patients in their sample were unlikely to present at a particular physician's practice because of the latter's drug preference. The strategy adopted by Hebert et al. (2013) is explored in greater detail in the section below and follows a similar approach involving a restriction on the study period. The second strategy involved ruling out unmeasured confounding due to the complexity required for it to be plausible (*n* = 1). Hebert et al. (2013) argued that unmeasured confounding would imply that patient behavior would have to be complex such that they chose where to live based on local ACEI prescription rates. Lastly, studies also articulated a specific mechanism that allowed unmeasured confounding to be ruled out (*n* = 4).

Nine falsification tests were performed across nine studies. Five studies provided the descriptive statistics of their baseline covariates at each level of their IV. Two performed baseline hypothesis testing. Two reported standardized differences on baseline characteristics. One study reported scaled bias component plots. A bias component plot is a graphical method that compares the bias that would be incurred in an instrumental analysis versus noninstrumental analysis (i.e., modeling the effect of the exposure on outcome directly) had a measured covariate been omitted from adjustment (Davies et al., 2017); scaling may be introduced using the strength of the IV (Jackson & Swanson, 2015). An assumption of bias component plots is that if omitting any measured covariate does not result in a bias in the IV estimate, then the presence of unmeasured covariates will also not result in bias. Two studies conducted sensitivity analyses on the suspicion that the exchangeability assumption was more likely to hold in one subgroup, and thus differences in subgroup results in theory should be attributed to the exposure (method of population stratification). For example, Lei et al. (2020) performed a sensitivity analysis by excluding veterans in assisted living residence to reduce the influence of poor health conditions, which may be an unmeasured confounder of the IV‐outcome relationship as veterans in may choose to move due to health reasons. Two studies used a negative control outcome to detect unmeasured confounding bias. Negative control outcomes are assumed to not be caused by the exposure but may still share common causes with the IV. Associations between the IV and the negative control outcome may signal a violation of exchangeability (Davies et al., 2017). Both Lind et al. (2021) and Thunell et al. (2022) created their negative control outcomes by simply restricting their follow‐up period. This was based on the reasoning that there would unlikely be an effect of their exposure of interest on incident dementia within a short time duration. One study used the Sargan–Hansen overidentification test. Walker et al. (2020) believed that their seven‐point ordinal IV may have led to overidentification and performed the Sargan–Hansen test on two dichotomized versions of their IV. The overidentification test (Hansen, 1982; Sargan, 1958) is mainly performed when an investigator uses multiple IVs and there is a risk that there are more IVs than what is necessary to identify causal effects (overidentification). Under the assumption that all IVs are valid, the test assesses whether one or more of the IVs violate the exchangeability assumption but not specifically which (Bollen, 2012). Lastly, the subgroup method used by Nguyen et al. (2016) and the inequality test used by Walker et al. (2020), described earlier in the sections above, also test the falsifiability of exchangeability.

### 3.5 Strategic study designs

We explored study design strategies that supported the validity of an IV and report two noteworthy case studies. The first strategy involved capitalizing on prior subject‐matter knowledge of seminal papers that may have influenced clinical practice. Hebert et al. (2013) restricted their study period to the years prior to the publication of two observational studies showing an association between use of centrally‐active angiotensin‐converting enzyme inhibitors (CA ACEI) compared to non‐CA ACEIs on cognitive decline and dementia risk. In doing so, the exclusion restriction and exchangeability assumptions could arguably have been met because ACEIs would have been viewed as an undifferentiated class during that period. Thus, the physician's preference for the type of ACEI should not be influenced by unmeasured patient characteristics (i.e., confounders) nor lead to differences in future care and treatment (i.e., a secondary pathway/mechanism by which ACEI preference may lead to dementia risk).

The second strategy involved leveraging subject‐matter knowledge to define IVs such that it is possible to identify and exclude participants for whom the IV is known to have no influence on the exposure variable. Nguyen et al. (2016) analyzed a subgroup of participants with less than 12 years of education because the schooling policies used in the operationalization of their multiple IVs were only relevant for the years spanning pre‐tertiary education. In addition to helping to meet the relevance assumption, the advantage of this design was highlighted earlier where they were able to apply a falsification test for both exclusion restriction and exchangeability in the subgroup of participants with postsecondary education.

## 4 DISCUSSION

We systematically reviewed the subject‐matter argumentation, falsification test, and study design strategies of 12 clinical studies of dementia and neurodegenerative disease. All studies made at least one subject‐matter argument or falsification test, indicating that justifying the validity of an IV was accepted as a fundamental requirement.

Overall, however, the practice of providing subject‐matter arguments and conducting falsification tests for all three assumptions in an individual study was not commonplace. The use of falsification tests was more frequent than subject‐matter arguments. Justification of the relevance assumption was conducted unanimously with a preference for reporting the quantitative evidence of the strength of the IV. Justification for exclusion restriction was least commonly conducted and there was a majority preference for subject‐matter argumentation. Justification for exchangeability was most frequently approached with falsification testing. All studies did acknowledge that IV analysis rests on assumptions either in their introduction or methods sections, but only a minority explicitly stated all three assumptions (Supporting Information 3). It was not always clear if assumptions were omitted or combined into a single assumption. Some studies had assumed there were only two IV assumptions. This practice may explain the relative lack of attention toward justifying the exclusion restriction assumption. The tendency to combine the exclusion restriction and exchangeability assumptions is consistent with prior research on reporting practices (Swanson & Hernán, 2013) and may reflect that, statistically, the two assumptions have shared falsification tests (Labrecque & Swanson, 2018).

We observed a large discrepancy in the uptake of IV between preclinical and clinical research during our abstract screening. We postulate that some potential barriers to the uptake of IV methodology in clinical research into dementia and neurodegenerative disease may be the uncertainty over how to define a valid IV with clinical data, lack of knowledge on suitable large observational datasets (Singh et al., 2018), unfamiliarity with the methodology, and concerns that peer review may be met with heightened skepticism (Pullenayegum et al., 2016). Concerning data, this systematic review outlined several options that are demonstrably suitable for IV analysis, particularly within Japan, United States, and United Kingdom (Table 2). Regarding peer review, given that IV research may be uncommon, this concern is understandable as reviewers themselves would be unfamiliar. Skeptical reviewers may be assuaged by presenting evidence from this review that IV analysis can be a suitable method to investigate causal effects in dementia and neurodegenerative disease—although we would argue that this is conditional on having a justifiably valid IV.

The strength of our review is that it contributes additional insights on top of previous reviews (Chen & Briesacher, 2011; Davies et al., 2013; Swanson & Hernán, 2013). We applied qualitative methods and coded text data to cover various types of subject‐matter argument approaches, covered a wider selection of falsification tests, and discussed innovative design strategies. We also improved upon a rating scale by Chen and Briesacher (2011) by awarding subject‐matter arguments and falsification tests separately. This modification reflects real‐world practice where studies may not necessarily perform both. Our IV validation appraisal tool may be a useful starting point for researchers and reviewers of research to evaluate whether there is a convincing case for a valid IV. We have included the extracted verbatim text in supplemental materials to be transparent with our approach. Codes are dependent on the investigators’ interpretation and we minimized this bias by including the perspectives of academic clinicians (TY, JH, and JPT) and statistical methodologists (SH, MDT, and NL).

Our review had limitations. We were not able to provide complete coverage of every possible subject‐matter argument or falsification test as we were sampling from a distinct subject area. Our IV validation appraisal only scored based on the presence of attempts to validate an IV rather than whether the validation was robust and sound. Many of the subject‐matter arguments are in themselves subjective and may not be accepted by all critical reviewers even if described well by the authors. Fortunately, we did not detect any improper strategies, with the possible exception of the falsification test for exclusion restriction by Hikichi et al. (2016) whereby they assessed the statistical significance of the effect of the IV on the outcome after adjusting for the exposure. Baiocchi et al. (2014) argued that this is not a valid even if the three IV assumptions were true. It was also beyond the scope of our review to cover a fourth IV assumption that is strictly related to identifying a point estimate of a causal effect. Without making this fourth assumption, the three “core” IV assumptions only enable estimation of the upper and lower bounds on the average causal effect (Baiocchi et al., 2014). However, we argue that the three “core” IV assumptions discussed here must first be met before any discussion about effect identification should take place.

## 5 CONCLUSIONS

We conclude with practical recommendations below and some additional assumption‐specific considerations (Table 4) for justifying the validity of an IV in future clinical research into dementia and neurodegenerative disease.

1. Close collaborations between clinicians and statisticians during the design and analysis of IV studies to provide convincing subject‐matter arguments and propose appropriate falsification tests.
2. Explicitly describe all three assumptions individually and provide a description what each means to facilitate planning around the subject‐matter arguments and falsification tests needed. Keep exclusion restriction and exchangeability as distinct assumptions to allow for their respective subject‐matter arguments (Swanson & Hernán, 2013). Lengthy subject‐matter arguments could be added as supplemental material.
3. Causal diagrams may be utilized to give investigators a visual representation of their assumptions about the true causal structure, diagnose possible sources of bias, and identify a minimally sufficient adjustment set of variables. Frameworks for developing causal diagrams with domain experts (Rodrigues et al., 2022) and free software (Textor et al., 2011) are available.
4. Falsification tests that jointly test multiple IV assumptions may help researchers get an overall sense of whether their chosen IV is valid before delving into assumption‐specific tests.
5. Use expert knowledge to enact strategic study designs that help facilitate plausible justification of multiple IV assumptions.
6. Using Walker et al. (2020) as a guiding example of a study that incorporates subject‐matter arguments and falsification tests for all three assumptions.

**TABLE 4:** Additional assumption‐specific considerations to help guide the justification of a valid instrument.

Assumption	Main question	Additional considerations
Relevance	Does the instrument influence the exposure or treatment?	Are there prior quantitative or qualitative studies that show an association between the IV and the exposure of interest? Is it possible to give a plausible account for how and why the IV might influence the exposure? Do report the statistic used to represent the strength of the IV and its observed value?
Exclusion restriction	Could the instrument cause the outcome through other means beside the exposure?	The assumption does not limit itself to contexts where the IV directly causes the outcome; any other mechanism that is not the exposure could violate the assumption. Considering your IV, exposure, and outcome combination, is it possible to give a plausible account of another way for the IV to influence the outcome that has nothing to do with the exposure? Consider the follow‐up time in your study period, were there possible changes in practice or guidelines that may create an alternative mechanism for the IV to have an influence on the outcome? Is it possible to identify a subpopulation where the IV does not cause the exposure for falsification testing?
Exchangeability	Are there any common causes of the instrument and outcome that could provide an alternate explanation for the associations observed?	Would explanations for unmeasured confounding involve implausibly complex mechanisms? Is there a subpopulation where the exchangeability assumption may be more likely to hold? Consider adding table of baseline descriptive statistics for each IV level. Hypothesis testing to show differences in baseline covariates between IV levels is subjected to sample size. Standardized mean differences or bias component plots may be good supplements to the table. Consider drawing a causal diagram to assess if confounding bias from unmeasured covariates may be mitigated by adjusting for what is observed in the available data. If this is possible, then the exchangeability assumption may be relaxed and investigators could satisfy the conditional exchangeability assumption such that the IV and outcome are assumed to be unconfounded once certain covariates are adjusted. Can a suitable negative control outcome be used in the available data?

Abbreviation: IV, instrumental variable.

## AUTHOR CONTRIBUTIONS

Shaun Hiu contributed to the conceptualization and design of the review, screened studies for eligibility, extracted and interpreted the data, drafted the manuscript, and reviewed the manuscript. Tingting Yong contributed to the design of the review, screened studies for eligibility, extracted and interpreted the data, and reviewed the manuscript. Jahfer Hasoon contributed to the design of the review, interpreted the data, and reviewed the manuscript. M. Dawn Teare contributed to the design of the review, interpreted the data, and reviewed the manuscript. John‐Paul Taylor contributed to the design of the review, interpreted the data, and reviewed the manuscript. Nan Lin contributed to the design of the review, interpreted the data, and reviewed the manuscript.

## CONFLICT OF INTEREST STATEMENT

All authors declare no conflicts of interest.

### PEER REVIEW

The peer review history for this article is available at https://publons.com/publon/10.1002/brb3.3371.

## Supporting information

Supplemental Material 1: Search terms and set of clinical outcomes of interest

Supplemental Material 2: Verbatim text data used to form subject‐matter argument descriptors

Supplementary Material 3: Verbatim text of studies’ descriptions of the IV assumptions

## Data Availability

The data that support the findings of this study were obtained from publicly available sources and are available from the corresponding author upon reasonable request.
