# Supplementary material for: Instrumental variables in real‐world clinical studies of dementia and neurodegenerative disease: Systematic review of the subject‐matter argumentation, falsification test, and study design strategies to justify a valid instrument
Source: Brain Behav. 2024 Jan 6;14(1):e3371. doi: 10.1002/brb3.3371 (PMC10771230; doi:10.1002/brb3.3371)
Supplement: Supplementary file 1 — Supplemental Material 1: Search terms and set of clinical outcomes of interest [file BRB3-14-e3371-s003.docx]

**Supplemental Material 1: Search terms and set of clinical outcomes of interest**

| **Search terms** |
| --- |
| (“instrumental variable” OR “Mendelian randomization”) AND (“dementia” OR “vascular dementia” OR “neurocognitive disorder” OR neurodegenerat* OR Parkinson OR lewy OR Alzheimer OR Huntington OR frontotemporal OR FTD OR “multiple sclerosis” OR “multiple system atrophy” OR “motor neuron disease” OR “amyotrophic lateral sclerosis” OR “progressive supranuclear palsy” OR “chronic traumatic encephalopathy” OR “posterior cortical atrophy” OR “corticobasal degeneration”) |

| **Set of clinical outcomes of interest** |
| --- |
| dementia, neurodegenerative disease, mortality, hospitalisation, institutionalisation, serious adverse outcomes (e.g., myocardial infarction, stroke, pneumonia, septicaemia), medication adherence, cognition, neuropsychiatric symptoms, and biomarkers of neurodegeneration |
