# Supplementary material for: Instrumental variables in real‐world clinical studies of dementia and neurodegenerative disease: Systematic review of the subject‐matter argumentation, falsification test, and study design strategies to justify a valid instrument
Source: Brain Behav. 2024 Jan 6;14(1):e3371. doi: 10.1002/brb3.3371 (PMC10771230; doi:10.1002/brb3.3371)
Supplement: Supplementary file 2 — Supplemental Material 2: Verbatim text data used to form subject‐matter argument descriptors [file BRB3-14-e3371-s001.docx]

**Supplemental Material 2: Verbatim text data used to form subject-matter argument descriptors**

**Relevance**

|  | **Prior quantitative or qualitative evidence** | **Explanation of mechanism how IV influences exposure** |
| --- | --- | --- |
| Burke 2022 | “As distance to postacute care has been shown to be a strong determinant of postacute care choice,^15–17^ we used the difference in the distance between the centroid of the zip code of the patient's residence and the nearest HH agency and SNF within their HRR” (p. 499).  “First, qualitative studies demonstrate that distance to a HH provider is an important criterion for patients and caregivers when choosing among agencies.^36^ In fact, location of HH agency is the main criterion hospital discharge planners use to create a list for patients to choose from prior to discharge, and is how HH compare is designed (entering the patient's address to find nearby home care agencies)” (pp. 502-503). | “Unlike SNF, the business address of a HH agency is not where care is delivered, raising the question of why distance to the business address of the agency would predict treatment choice… [The] business office is likely to be proximate to where care is delivered. As most referrals are driven by the location of the business address, not providing care in that area would not be in the economic interest of the home care agency. The business address may also serve relevant administrative functions that make it important for it to be proximate to the areas of care delivery by HH agency staff. For example, HH supplies would be delivered to the business address, making it unlikely the business address would be far from where care is delivered. New referrals, billing questions, and other administrative functions are also consolidated at the business address, potentially requiring HH staff to travel to the office” (pp. 502-503). |
| Joyce 2018 | “First, we assume that differential distance is negatively correlated with admission to a facility with an SCU. We base this assumption on a large body of literature demonstrating that distance is a strong driver of provider choice, including nursing homes (Hartmaier et al. 1994; McClellan, McNeil, and Newhouse 1994; Phillips and Morris 1997; Hirth et al. 2014; Paksarian et al. 2016; Gadbois, Tyler, and Mor 2017). Thus, the greater the distance that an individual must travel to a nursing home with an SCU relative to a nursing home without an SCU, the less likely that individual is to be admitted to a facility with an SCU” (p. 3663). |  |
| Lei 2020 |  | “A change in residence was highly correlated with COC (partial F1,139= 384, P <0.01; Appendix 3, Supplemental Digital Content 1, http:// links.lww.com/MLR/C98) possibly because veterans who moved more than 10 miles may have needed to change their “usual” providers, resulting in lower COC” (p. 990). |
| Lind 2021 |  | “We hypothesized that county-level WMV utilization rates would be predictive of AWV utilization because the WMV measures area-level uptake of preventive care and provider willingness to conduct preventive care visits for Medicare beneficiaries, but the WMV utilization rates would not be associated with the probability of receiving a new dementia diagnosis conditional on other factors that can be observed” (p. 195). |
| Reynolds 2020 | “In this case, we used choice of individual medications for the same condition with similar efficacy and tolerability as an instrument. We knew, from prior work, that individual medications are strongly associated with OOP costs, thereby satisfying the first instrumental variable assumption.^3^” (p. e1417). |  |
| Sato 2021 | “In snowy areas, there are fewer opportunities to exercise outside, and older people who fear falling due to snow may avoid going to indoor exercise facilities” (p. 2). |  |
| Walker 2020 | “Physicians’ prescribing preference has been proposed as an instrumental variable in pharmacoepidemiology.^20–24^ It meets the instrument conditions, as (1) it is associated with the prescription issued by the physician…” (p. 853) |  |

**Exclusion Restriction**

|  | Other mechanisms unlikely due to nature of outcome | Other mechanisms unlikely due to nature of IV | Reasonable assumption given nature of exposure | Other mechanisms unlikely but without further elaboration |
| --- | --- | --- | --- | --- |
| Lind 2021 | “Fifth, our results may be limited by potential productivity spillovers, in that providers in areas with high rates of preventive visits like the AWV and WMV may get better at delivering preventive care, and in turn, may then have a healthier group of Medicare beneficiaries relative to areas with lower rates of preventive visits.^38^ However, the decades-long time course for the development of dementia suggests this is unlikely to be a substantial source of bias” (p. 201). |  |  |  |
| Nguyen 2016 |  | “We assume the IV effects are fully mediated by education, that is, no pathway exists between instruments and dementia that does not involve education. A pathway that we worry about is through intelligence or cognitive function. This is not plausible for CSLs or state school characteristics, which are identified from state and year of birth, thus avoiding correlations with any individual-level variables” (p. 74). |  |  |
| Reynolds 2020 |  |  | “While the exclusion restriction cannot be tested directly, it is reasonable within these groups of medications that the primary determinants of adherence—efficacy and tolerability—are similar across our selected medications” (p. e1417). |  |
| Sato 2021 |  | “Residency in a snowy area could be an acceptable IV for physical activity, given that snowfall hinders older adults from physical activity but does not directly affect dementia onset [14, 15]” (p. 2)  “Assumption (ii) can be violated if the IV affects the outcome through an alternative pathway (i.e., other than through the treatment pathway). However, it is difficult to think of a plausible mechanism by which living in a snowy area would directly increase the risk of dementia” (p. 8). |  |  |
| Thunell 2022 |  | “In contrast, our instrument accounted for changes in rates of county-level visits, and among similar sized areas. While differences across counties in level of visits in a year may be correlated with individuals’ AWV receipt and dementia diagnoses, for example through generally better (or worse) access to healthcare, changes in AWV visits measure growth or decline from year to year is less likely to be associated with dementia diagnoses, except through an individual’s AWV” (p. 6). |  |  |
| Walker 2020 |  |  |  | “(2) it is unlikely to relate to the patient’s risk of dementia other than through the prescription issued” (p. 853). |

**Exchangeability**

|  | **Unmeasured confounding implausible due to nature of study period** | **Unmeasured confounding unlikely due to the required complexity** | **Provision of mechanism why unmeasured confounding implausible** |
| --- | --- | --- | --- |
| Burke 2022 |  |  | “In particular, instrumental variable methods may better address important unmeasured factors in our data, such as caregiver support. While caregiver support may play a large role in hospital discharge decision making, the presence of a caregiver is unlikely to confound our findings unless caregiver support is distributed in such a way that it is correlated with differential distance—for example, if patients who live closer to SNF than HH are more or less likely to have caregiver support. Although we are not aware of any data that speak to this, it does not seem plausible that caregiver availability and differential distance to postacute care provider type are strongly correlated. However, we are unable to directly test for the relationship between any unobserved factors and the instrument, a limitation of this analytic approach” (p. 502). |
| Hebert 2013 | “Consequently, clinicians are unlikely to select an ACEI on the basis of its pharmacodynamic properties or the cognitive characteristics of the patient, which reduces the likelihood of selection bias or confounding by indication. This was particularly true during the study period, which preceded the publication of the observational studies” (p. 642). | “First, like all observational studies, the results are subject to unobserved confounding, although in the case of the IV analysis, the confounding would have to be complex: high-risk patients would have to systematically choose clinicians according to the clinician’s ACEI prescribing behavior for other patients or choose where to live on the basis of the ACEI use of other Medicare beneficiaries in the area” (p. 647). | “For this IV, we relied on the fact that there are no well-defined differences between ACEIs; some researchers refer to all ACEIs as an undifferentiated class of medication.^13^ Physicians generally prescribe the ACEI with which the physician or patient is most comfortable, regardless of whether that ACEI is CA or not. If a physician has a preference for a particular ACEI, then the ACEI prescribed to the prior patient is likely to be correlated with the ACEI prescribed to the given patient, but this choice of ACEI is unlikely to be correlated with the given patient’s cognitive health because the physician was not considering the given patient’s health when he or she chose an ACEI for the prior patient. Thus, we used the type of ACEI prescribed to the prior patient as an instrument for the ACEI prescribed to the given patient” (p. 645). |
| Joyce 2018 |  |  | “Because distance is important toward predicting nursing home choice and an individual chooses where to live without regard as to whether nearby nursing homes have an SCU, the identifying assumption is that the instrument will be correlated with the selection of a nursing home with an SCU but independent of patient-specific measures that would determine selection” (p. 3659).  “A second assumption is that the instrument is uncorrelated with the error term in the second stage estimation. A violation of this assumption could occur if individuals choose their residence based on the SCU status of the nearest nursing home, or nursing homes with SCUs chose their location based on the characteristics of the local population differently than nursing homes without SCUs… However, the second assumption can only be evaluated on the set of observables measured in the MDS and census. Evidence suggesting that differential distance is independent of the set of observed characteristics leads to greater confidence that it is also independent of unobserved confounders, although this is not possible to prove.” (p. 3663). |
| Walker 2020 | “(3) physicians’ prescribing preference is unlikely to share a cause with the patient’s outcome because patients have relatively little choice over which physician they see or knowledge of their physicians’ preferences for antihypertensive drug classes.^22^ The last condition is likely to hold in the UK setting used for this study because prior to 5 January 2015, patients were required to live within a practice’s boundary area to register at that practice, which limited their choice of physician.^25^” (p. 853). |  | “(3) physicians’ prescribing preference is unlikely to share a cause with the patient’s outcome because patients have relatively little choice over which physician they see or knowledge of their physicians’ preferences for antihypertensive drug classes.^22^” (p. 853). |
