# Supplementary material for: Instrumental variables in real‐world clinical studies of dementia and neurodegenerative disease: Systematic review of the subject‐matter argumentation, falsification test, and study design strategies to justify a valid instrument
Source: Brain Behav. 2024 Jan 6;14(1):e3371. doi: 10.1002/brb3.3371 (PMC10771230; doi:10.1002/brb3.3371)
Supplement: Supplementary file 3 — Supplementary Material 3: Verbatim text of studies’ descriptions of the IV assumptions [file BRB3-14-e3371-s002.docx]

**Supplementary Material 3: Verbatim text of studies’ descriptions of the IV assumptions**

| **Study** | **Verbatim text describing IV assumptions** | **Assumptions described** |
| --- | --- | --- |
| Burke 2022 | “Instrumental variable analyses approximate random assignment of patients to treatment groups based on an instrumental variable, which strongly influences type of treatment received but is not linked to the outcome or other factors associated with the outcome, and is randomly distributed across the population of interest” (p. 499). | Relevance described  Exclusion Restriction and Exchangeability combined into one assumption. |
| Hebert 2013 | “The key to an IV analysis is finding a variable (the instrument) that, much like the random assignment in a randomized trial, divides patients into two groups that differ by nothing except the treatment of interest. If two groups differ by nothing but the treatment they received, then any difference in outcomes can be reasonably and causally attributed to differences in treatment” (p. 645). | Relevance described  Exclusion Restriction not described  Exchangeability described. |
| Hikichi 2016 | “A valid instrument requires that it be associated with the treatment, but not directly affect the outcome” (p. E6917). | Relevance described  Exclusion Restriction and Exchangeability combined into one assumption. |
| Jayadevappa 2019 | “We used an instrumental variable approach to address unmeasured bias, relying on an instrumental variable that is associated with the likelihood of receiving a type of treatment but is independent of diagnosis of Alzheimer disease or dementia.^30^ An appropriate instrument is one that is associated with the exposure (ADT treatment) but not with the outcome(s)” (p. 3). | Relevance described  Exclusion Restriction and Exchangeability combined into one assumption |
| Joyce 2018 | “[The] identifying assumption is that the instrument will be correlated with the selection of a nursing home with an SCU but independent of patient-specific measures that would determine selection” (p. 3659). | Relevance described  Exclusion Restriction not described  Exchangeability described |
| Lei 2020 | “We used an instrumental variable approach to address the endogeneity of COC and to identify the causality between COC and hospitalization. The instrument was whether the veteran changed residence (longitude/latitude) by more than 10 miles in FY 2014 (5.4% of cohort). A change in residence was highly correlated with COC (partial F1,139= 384, P <0.01)” (p. 990).  “The instrumental variable approach also assumed that a change in residence was not correlated with the hospitalization outcomes, except through COC… In addition, there should not be any mutual confounders between the instrument and outcomes.” (p. 990). | Relevance not described explicitly, but implied via an analysis  Exclusion Restriction and Exchangeability described |
| Lind 2021 | “The validity of this instrument hinges on two conditions: (a) how well it predicts individual AWV utilization and (b) whether it can be validly excluded from the main equation (ie, the WMV is not a confounder). Specifically, this implies that the WMV is unrelated to the likelihood of receiving a new dementia diagnosis after controlling for covariates” (p. 195). | Relevance described  Exclusion Restriction not described  Exchangeability described |
| Nguyen 2016 | “To yield valid results, all instruments must meet three main assumptions. First, the instruments must predict the exposure. Second, the instruments must affect the outcome only through the exposure. Finally, the instruments must not share unmeasured common causes with the outcome” (p. 73). | All assumptions described individually |
| Reynolds 2020 | “Instrumental variable approaches can be used to estimate the causal effect of OOP cost on adherence even when not directly adjusting for unmeasured confounders if 2 key assumptions are met: (1) the instrument is strongly associated with the exposure of interest and (2) there is no relationship between the instrument and the outcome, other than through the key exposure (the exclusion restriction)” (p. e1417). | Relevance described  Exclusion Restriction described  Exchangeability not described |
| Sato 2021 | “Briefly, if there is an appropriate IV (i.e., a variable that exogenously determines treatment levels, thus assuring exchangeability between treatment and control groups and thereby simulating RCTs), this can eliminate reverse causation bias and enable causal inferences for the association between physical activity and dementia. Residency in a snowy area could be an acceptable IV for physical activity, given that snowfall hinders older adults from physical activity but does not directly affect dementia onset” (p. 2).  “As noted above, the IV method needs to satisfy three assumptions: (i) the relevance condition, (ii) the exclusion restriction, and (iii) marginal exchangeability. For assumption (i), we confirmed that residency in a snowy area was negatively associated with physical activity… Assumption (ii) can be violated if the IV affects the outcome through an alternative pathway… Assumption  (iii) can be violated if the IV shares causes with the outcome“ (p. 8). | All assumptions described individually |
| Thunell 2022 | “Instrumental variables (IV) estimation is an advanced statistical method that attempts to isolate exogenous variation in a potentially endogenous predictor, in our case individual AWV receipt. The key reason for using the IV approach is that there are unobserved confounders that intervene in the relationship between the outcome (dementia diagnosis) and the predictor. For an instrument to be valid, it must be related to the outcome only through its relationship with the predictor. See Supplemental Digital Content Figure S2 for a visual representation of these relationships” (p. 3)  “As mentioned above, a key assumption is that, after controlling for observable confounders, the county-level change in AWV utilization is related to the likelihood of dementia diagnosis only through its impact on AWV use” (p. 3). | Relevance not described  Exclusion Restriction and Exchangeability combined into one assumption |
| Walker 2020 | “Instrumental variable analysis, which estimates the causal effect of an exposure on an outcome by using a third variable (the instrument), can be robust to confounding and reverse causation if certain assumptions are met (Figure 1). That is, the instrument must (1) be associated with the exposure of interest; (2) affect the outcome only through its effect on the exposure; and (3) have no common causes with the outcome (i.e., no confounders of the instrument–outcome association)” (p. 853) | All assumptions described individually |
